# Predicting outcomes of steady-state ^13^C isotope tracing experiments using Monte Carlo sampling

**DOI:** 10.1186/1752-0509-6-9

**Published:** 2012-01-30

**Authors:** Jan Schellenberger, Daniel C Zielinski, Wing Choi, Sunthosh Madireddi, Vasiliy Portnoy, David A Scott, Jennifer L Reed, Andrei L Osterman, Bernhard ∅ Palsson

**Affiliations:** 1 Bioinformatics and Systems Biology Program, University of California - San Diego, 9500 Gilman Drive, La Jolla, CA, 92093-0419 USA; 2 Department of Bioengineering, University of California - San Diego, 9500 Gilman Drive, La Jolla, CA, 92093-0412 USA; 3 Bioinformatics and Systems Biology Program, Sanford-Burnham Institute, 10901 North Torrey Pines Road, La Jolla, CA, 92037 USA; 4 Department of Chemical and Biological Engineering, University of Wisconsin Madison, 1415 Engineering Drive, Madison, WI, 53706-1607 USA

## Abstract

**Background:**

Carbon-13 (^13^C) analysis is a commonly used method for estimating reaction rates in biochemical networks. The choice of carbon labeling pattern is an important consideration when designing these experiments. We present a novel Monte Carlo algorithm for finding the optimal substrate input label for a particular experimental objective (flux or flux ratio). Unlike previous work, this method does not require assumption of the flux distribution beforehand.

**Results:**

Using a large *E. coli *isotopomer model, different commercially available substrate labeling patterns were tested computationally for their ability to determine reaction fluxes. The choice of optimal labeled substrate was found to be dependent upon the desired experimental objective. Many commercially available labels are predicted to be outperformed by complex labeling patterns. Based on Monte Carlo Sampling, the dimensionality of experimental data was found to be considerably less than anticipated, suggesting that effectiveness of ^13^C experiments for determining reaction fluxes across a large-scale metabolic network is less than previously believed.

**Conclusions:**

While ^13^C analysis is a useful tool in systems biology, high redundancy in measurements limits the information that can be obtained from each experiment. It is however possible to compute potential limitations before an experiment is run and predict whether, and to what degree, the rate of each reaction can be resolved.

## Background

**Figure 1:**
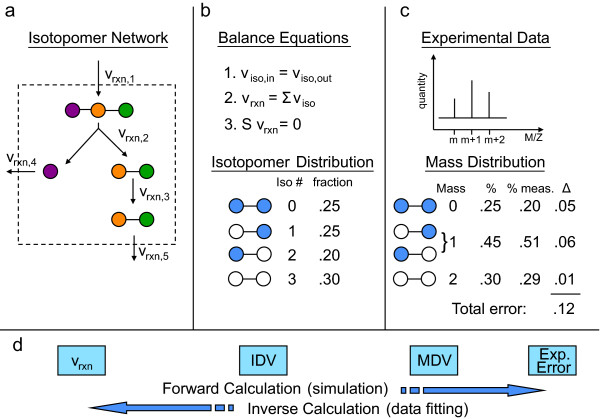
**Isotopomer Overview**. a) definition of the network, including carbon fates b) isotopomer balance equations - solving these equations yields the Isotopomer Distribution Vector (IDV) c) experimental data are compared to computed Mass Distribution Vectors (MDV) yielding experimental fit. d) two types of possible computations - the forward computation uses a flux distribution as input to compute the MDV, while the inverse problem attempts to find the flux distribution that minimizes the experimental discrepancy.

*In vivo *metabolic reaction flux data provides insight into the dynamic function of the cell [1-3]. One widely-used experimental method for measuring *in vivo *reaction fluxes is steady-state substrate ^13^C isotope labeling [4-6]. An overview of the general ^13^C methods is described in Figure 1. Isotopomers, or isomers created from inserting labeled isotopes (often ^13^C) at different positions in a molecule, provide a unique way to track the progress of carbon through a metabolic network. By measuring the enrichment for ^13^C in metabolite pools after growing on a ^13^C labeled substrate, inferences about the internal flux state can be made. The approach can be summarized as a data fitting problem between simulated and experimentally measured ^13^C labeled metabolite concentrations. An isotopomer model, describing the positional transfer of carbon atoms for all or a subset of reactions in the network, is used to simulate data (Figure 1a). For a specified carbon input label, an isotopomer model enables the calculation of an isotopomer distribution vector (IDV) corresponding to a particular simulated steady-state flux distribution (Figure 1b). Mass spectrometry (MS) experiments on ^13^C-labeled metabolites (e.g. macromolecules) generate fractional ^13^C enrichments from fragmented macromolecules, forming a mass distribution vector (MDV) (Figure 1c). The error between the measured MDV and the MDV corresponding to the simulated IDV summarizes how well the presumed flux distribution fits the ^13^C experiment. The flux distribution *v *that minimizes this error can be computed by solving a non-linear optimization problem. Simulating ^13^C enrichment given a flux distribution is computationally inexpensive; however, the inverse problem of calculating the flux distribution that best fits a ^13^C experiment is both of greater interest and significantly more computationally difficult (Figure 1d). A review of these methods and associated challenges can be found in [6-8].

There are several distinct sources of variability in a ^13^C experiment that limit the confidence with which particular reactions can be determined. First, due to experimental accuracy limitations and biological variability, uncertainty arises in the experimentally measured MDV. Second, due to alternate pathways present in metabolic networks, the mass balance equations underlying a metabolic steady-state are significantly under-determined [9]. While the full network flux distribution may not be resolved at high confidence by a given experiment, certain labeling patterns may resolve fluxes through certain pathways with greater confidence than other labeling patterns, as has previously been shown [4,10].

For a given n-carbon compound, there are 2*^n ^*possible ^13^C labeling states (as well as mixtures), and the choice of label is known to affect the ability to determine reactions fluxes [10]. As ^13^C methods are based upon computational modeling of isotopomer distributions, it is possible to computationally optimize the choice of substrate labeling pattern to enhance the information gained from an experiment. There are two primary motivations that drive such an endeavor. First, ^13^C experiments are expensive, so choosing the best experiment *a priori *is desirable. Second, we can assess the capability of the steady-state ^13^C labeling approach towards determining reaction fluxes in an unbiased manner. The issue of optimization of ^13^C labeling experiments has been addressed in the literature [4,10,11]. However, the use of flux sampling for optimal isotopomer experiment prediction has not been explored previously, and this approach presents several unique advantages over previous methods.

We describe a Monte Carlo sampling-based method for choosing the optimal substrate label, based upon the Constraint-Based Reconstruction and Analysis (COBRA) computational platform [12,13]. COBRA methods use manually-curated biochemical network reconstructions of known reaction stoichiometries and measurable nutrient uptake and secretion rates to define feasible ranges for internal reaction fluxes. Many of these reconstructions have been generated [14] and the procedure is well-established [13,15]. These models can be used for methods such as computing growth rates [16,17], predicting the effects of gene knockouts [16,18,19], predicting the endpoint of adaptive evolutions [20], and designing strains for industrial production [21,22]. A review of these methods can be found here [12,13,23]. Monte Carlo sampling of constraint-based metabolic models can be used to generate sets of biochemically feasible flux distributions that obey measured uptake and secretion rate constraints [24]. IDVs generated from these flux distributions in an isotopomer model can then be compared against simulated ^13^C data to evaluate the ability of the experiment to determine reaction fluxes. Monte Carlo sampling takes advantage of the speed with which IDVs can be simulated from putative flux distributions, making this approach suitable for large-scale analysis of *in silico *experiments.

A Monte Carlo sampling approach was implemented using a newly developed isotopomer model to evaluate the efficiency of different carbon labeling patterns toward determining reaction fluxes in *E. coli*. The dimensionality of simulated ^13^C data was calculated using singular value decomposition (SVD) for different substrate labeling patterns and compared to the number of undetermined dimensions in the network. ^13^C experiments were performed for three substrate labeling patterns to validate the prior theoretical analysis. The methods developed represent a flexible computational analysis that can be applied to various biological systems and experimental setups to estimate, *a priori*, the efficiency of isotopomer experiments in determining reaction fluxes.

## Results and Discussion

### Expanded Isotopomer Model

An isotopomer model was constructed in two phases. First, a central metabolic isotopomer model that accounts for 85 reactions including glycolysis, the TCA cycle, the pentose phosphate pathway, oxidative phosphorylation, pyruvate metabolism, and anaplerotic reactions was derived from the iJR904 *E. coli *reconstruction [16]. This initial model was equivalent in reaction content to commonly used isotopomer models for *E. coli *[25,26].

An expanded model was then constructed that includes both central and biosynthetic pathways. The iMC1010 metabolic network [19] was evaluated to determine which reactions can sustain non-zero fluxes during growth on glucose, acetate, or lactate when only certain by-products are allowed to be secreted (acetate, formate, D-lactate, pyruvate, succinate, glycerol, CO_2_, and ethanol). Blocked reactions, which must have zero net flux at steady state, were subsequently omitted from consideration. Groups of reactions that could be merged together without affecting model results (e.g. linear pathways) were combined in order to reduce the number of variables. Large sets of biosynthesis reactions that produce phospholipids, nucleotides, co-factors were also combined, since there no experimental measurements existed for these high-carbon metabolites. However, by-products resulting from high-carbon metabolite production (e.g. CO_2_, formate, succinate, fumarate, and pyruvate) that could enter back into the metabolic network were tracked. Of the original 932 reactions in the complete metabolic iMC1010 network, nearly a third were represented in the biosynthetic isotopomer model, either individually or as grouped reactions.

The final isotopomer model accounts for a total of 313 irreversible reactions, including 278 which track carbon. Inclusion of these additional pathways is likely important for accurate assessment of the flux-resolving power of ^13^C experiments both within and beyond central metabolism [7]. A complete listing of the reactions and metabolites in the biosynthetic network can be found in the Additional File 1.

### Monte Carlo Sampling Approach

To compute possible flux distributions of the *E. coli *model, the network was sampled using a Markov Chain, Monte Carlo (MCMC) sampling algorithm (see Methods). The steady-state mass balance and uptake rate constraints for the metabolic network create a convex hyperspace that contains all biochemically feasible steady-state flux distributions [27]. Monte Carlo sampling generates a set of flux distributions that are spread uniformly throughout the feasible space. The inclusion of ^13^C experimental data reduces the feasible space in which the true flux state must lie by requiring that the IDV calculated from the putative flux distribution must match the experimental data within error. While the space of feasible flux distributions depends only on reaction stoichiometry, the space of resulting simulated IDVs differs depending on the input substrate labeling pattern. Hence, different labeling patterns can have differing abilities to resolve each reaction flux.

**Figure 2:**
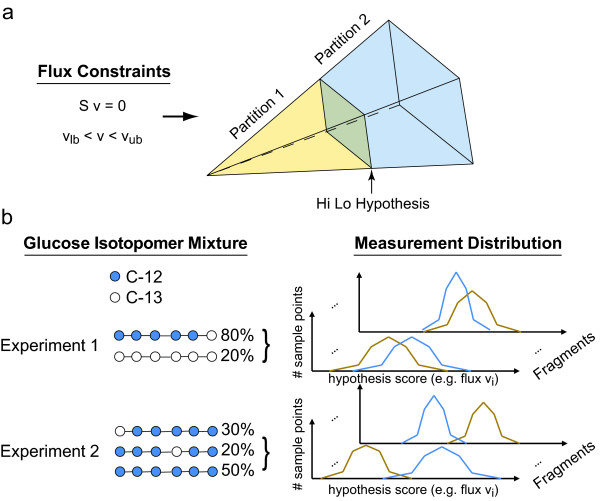
**Method Overview**. a) The space of flux distributions is partitioned in two parts corresponding to 'high' flux versus 'low' flux. A uniform random sample is drawn from the space and is also partitioned into partition 1 and partition 2. b) For each point in the space the distribution of experimental measurements is simulated. Hypothetical experiment 1 and experiment 2 with different glucose label mixtures produce different measurement distributions. The distributions from experiment 2 are more separated, indicating parameters of experiment 2 are more conducive for differentiating between the high and low partition.

Here, we used Monte Carlo sampling of flux distributions to analyze the degree to which reaction fluxes can be determined by steady-state ^13^C labeling experiments in terms of several possible experimental objectives. For example, one possible experimental objective is to determine whether a particular reaction has a flux above or below a specified value. For this objective, a well-designed labeling pattern would be one in which flux distributions that have an objective reaction flux greater than the specified value can be easily distinguished from flux distributions with an objective reaction flux less than the specified value. As seen in Figure 2, a hypothetical experiment 1 produces measurement distributions which overlap whereas experiment 2 shows greater separation. If one were interested in differentiating between the two partitions, experiment 2 would be much preferable. This method allows for the scoring of any label for any given experimental objective without first knowing the true cellular flux distribution *v*.

### **Generating and Evaluating **^13^**C Experimental Hypotheses**

An experimental hypothesis is defined as a partition of the sampled flux distribution set. While many possible hypotheses could be considered, two rational hypotheses were studied. The first case attempts to elucidate whether a reaction has high or low flux (hi-lo). The solution space is partitioned into all points with *v_j _>*threshold versus *v_j _<*threshold. A different hypothesis is generated for each reaction *j*. The threshold was chosen to be the median of all *v_j _*so that half of all points would be in each of the two partitions. The second set of hypotheses tested consisted of biologically relevant flux ratios. For each point the ratio of two reactions, *v_i_*/*v_j_*, was determined to be above or below some threshold that formed a partition.

Intuitively, a hypothesis score should be high if the isotopomer distributions coming from one partition are distinguishable from distributions in the other partition. While there are several ways of doing this, we chose a heuristic metric based on a Z-score, which is commonly used to determine the difference between two samples. A Z-score was calculated for each fragment (element) of the calculated MDV for each simulated flux distribution:

$$Z_{i}=\frac{\left|{\bar{x}}_{hi}-{\bar{x}}_{lo}\right|}{\sqrt{s_{hi}^{2}+s_{lo}^{2}+\sigma^{2}}}$$

where ${\bar{x}}_{hi}$ and ${\bar{x}}_{lo}$ are the average fragment enrichments for the upper and lower partitions, respectively, $s_{hi}^{2}$ and $s_{lo}^{2}$ are the variances of fragment enrichments for the upper and lower partitions, respectively, and the *α *is a constant equal to 0.014. *α *is on the order of magnitude of the uncertainty in measurements. This slight modification to the standard Z-score puts a lower bound on the expected experimental variation. The Z-score of each fragment is added together to give the Z-score of the experiment.

$$Z=\underset{i\in fragment}{\sum }Z_{i}$$

Using this approach, candidate flux states were sampled uniformly and experimental hypotheses tested. Z-scores were calculated for the hi-lo hypothesis corresponding to 1) individual reactions 2) reaction ratios and 3) two 'random' reactions or ratios. Random hypotheses were tested to estimate the level of noise associated with the set of flux distributions. Raw and normalized Z-scores are given in the Additional File 1. Z-scores varied from the level of noise to a maximum of *>*20-fold the level of noise.

**Figure 3:**
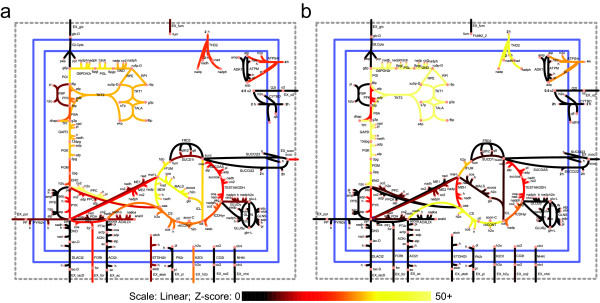
**Simulated Z-Scores**. Two possible glucose label patterns show different strengths in evaluating different parts of the network. Brighter colors indicate more easily determined fluxes. a) [1-^13^C] glucose Z-scores illustrates flux determinability with 100% [1-^13^C] glucose. b) [6-^13^C] glucose Z-scores shows the same network evaluated with [6-^13^C] glucose. It is observed, for example, that [6-^13^C] glucose is predicted to elucidate the pentose phosphate pathway more easily, while [1-^13^C] glucose better elucidates the glyoxylate shunt.

**Figure 4:**
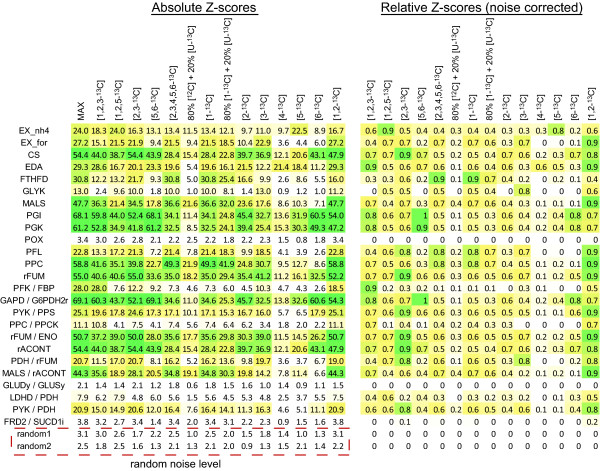
**Computational Evaluation of Glucose**. Potential glucose labels are evaluated based on both absolute Z-scores and Z-scores normalized with respect to the labeling pattern with the highest score. Glucose labels are listed on top including hypothetical labeling patterns (e.g. [1,2,3-^13^C]) and commercially available labels (e.g. [1-^13^C]). [U-^13^C] = uniform labeled and [^12^C] = unlabeled. Reaction and reaction ratio hypotheses are listed on the left. The 'random' hypotheses, as described in the methods, shows the level of noise.

To illustrate the differences in label-dependent reaction resolving capacity, two sets of Z-scores corresponding to [1-^13^C] glucose and [6-^13^C] glucose are plotted in Figure 3. Lighter colors indicate higher Z-scores and ease of measurement. In this case, [6-^13^C] glucose scores higher at measuring the pentose phosphate pathway and most of lower glycolysis, whereas [1-^13^C] glucose glucose scores much higher at measuring the glyoxylate shunt. The results suggest that there is no single label that yields a high score for all experimental objectives. For example, the exchange of formate (EX_for) could be easiest measured with a [1,2-^13^C] glucose; however, this labeling pattern is bested by [1-^13^C] glucose for the measurement of reaction formyltetrahydrofolate deformylase (FTHFD) (Figure 4). This non-universality of labels is in line with expectations, as it has been previously shown that the choice of labels can affect the flux resolution. For many reactions, the best experiment that could be performed involves hypothetical (non-commercially available) labels. One example is the ratio of phosphofructokinase (PFK) flux to fructose bisphosphate aldolase (FBA) flux. The best label for determining this ratio is [1,2,3-^13^C] glucose (Z = 28.0), which gives a much higher Z-score than the best commercially available label, [1,2-^13^C] glucose (Z = 18.5). Thus, there may be motivation to synthesize compounds with more complex labeling patterns than commonly used. Additionally, there are certain reactions which are predicted to be difficult to measure with any labeling pattern. For example, the Z-scores for each possible labeled glucose substrate for the reaction pyruvate oxidase (POX) all lie within the level of noise, as determined by the comparison with random hi-lo experiment Z-scores.

**Figure 5:**
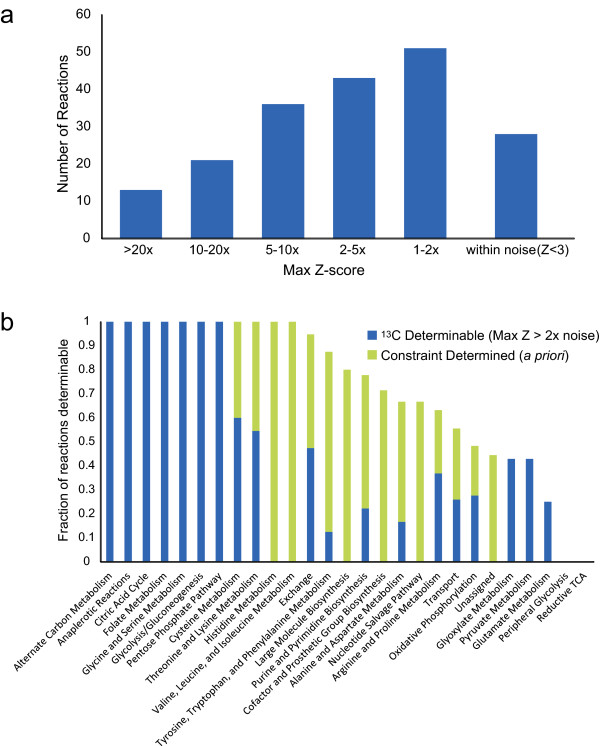
**Pathway Analysis of Optimal ^13^C Experiments**. a) The maximum Z-score of all labeling patterns for each non-fixed reaction was found. Notably, greater than 50% of reactions have no experiment with a predicted Z-score greater than twice the noise level. This indicates that many reactions will be difficult to elucidate. b) Reactions separated by subsystem. Reactions with fluxes fixed by constraints are termed "constraint determined." The reactions with maximum Z-scores at least twice the noise level were termed "^13^C determinable." The difference between determinable/determined fluxes and 1 is the fraction of reactions that cannot be determined through either ^13^C experiments or constraints.

In addition to label-specific reaction flux elucidation properties, ^13^C experiments show a clear pathway bias regardless of labeling pattern. The maximum Z-score of all labeling patterns was found for each reaction, giving a metric for the maximum potential for reaction flux determination using ^13^C-labeled glucose (Figure 5A). Then, the fraction of reactions that had a maximum potential at least twice the noise level was found for each subsystem (Figure 5b). Reactions that were stoichiometrically fixed by the measured constraints on acetate, glucose, D-lactate, oxygen, growth rate, and ATP maintenance were also categorized by subsystem. Stoichiometrically fixed (i.e. constraint-determined) reactions have a confidence interval of zero, and thus are label-independent and receive no additional knowledge from ^13^C experiments. It was found that histidine, valine, leucine, and isoleucine metabolism fluxes are completely identified solely based on the flux constraints. On the other hand, prior constraints fix none of the fluxes in central carbon metabolic systems such as glycolysis, citric acid cycle, pentose phosphate pathway, and anaplerotic reactions; however, fluxes in these pathways are all predicted to be identifiable with a ^13^C experiment using optimal labeling patterns for each reaction. This result is expected as these identifiable pathways are the typical pathways being studied using ^13^C analysis. Other pathways, such as cysteine, threonine, and lysine metabolism, are completely identifiable through a combination of prior stoichiometric constraints combined with well-chosen ^13^C experiments. However, many of the remaining subsystems have a fraction of reactions that cannot be determined using any ^13^C-labeling pattern of glucose. In particular, no additional information can be obtained from ^13^C-labeled glucose experiments about certain biosynthetic pathways, nucleotide salvage pathways, reductive citric acid cycle reactions, and certain alternate pathways peripheral to glycolysis, such as an alternate pathways from DHAP to D-lactate. Measuring metabolites other than amino acids may give more information on these pathways. Note that in this discussion of identifiability, the Z-score metric indicates that an experiment can significantly reduce the confidence interval of a particular reaction but does not specifically predict the value of the confidence interval. Confidence intervals are directly calculated for experimental data sets in a later section and compared to the Z-scores for the same labeling patterns.

### Dimensionality of Isotopomer Data

**Figure 6:**
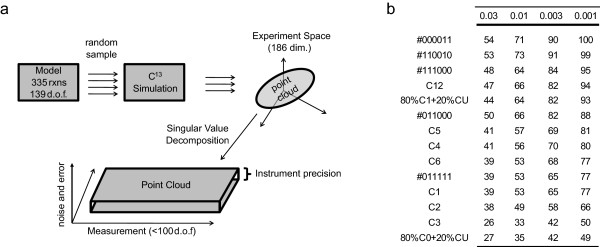
**Data Dimensionality with SVD**. The linear dimensionality of experimental data space is measured with Singular Value Decomposition. a) The E. coli model has 313 reactions and 139 degrees of freedom. The isotopomer fragments were computed for a random sample of flux distributions and plotted in the 186 dimensional space of simulated measurements. The upper bound on the number of degrees of freedom in this space was determined by singular value decomposition on the samples. The number of singular values was counted until the magnitude of the next singular value fell below the instrument threshold. b) The number of significant singular values at different levels of experimental error is tabulated for various labeling patterns, with values ranging from 26 to 100.

The Monte Carlo sampling approach enables the determination of the dimensionality of simulated ^13^C experiments for a particular substrate labeling pattern. The dimensionality gives an indication of the degree to which a particular substrate labeling pattern can specify the free dimensions inherent in a network structure, given a set experimental error. In an extreme case, if all the data falls on one point (zero dimensions), no additional information is given from the data. Similarly, a dimensionality of one indicates that the data can specify one degree of freedom. Singular value decomposition (SVD) is a data reduction technique that allows the estimation of data dimensionality (Figure 6a). A data matrix *M *of size (*n_fragments _*x *n_points_*), consisting of all sample points generated from Monte Carlo Sampling, is decomposed into *M *= *U *· Σ · *V ^T ^*where *U *and *V *are orthonormal bases and Σ is a diagonal matrix containing singular values in descending order. The singular values are effectively weightings that describe the information content of the corresponding vectors in *U *and *V *towards reconstructing the full matrix *M*. A partial reconstruction of *M *is possible by taking only a subset of the singular values greater than some threshold. These thresholds have a direct interpretation as the uncertainty with which a data point can be measured. For example, a threshold cutoff of 0.01 indicates that the remaining uncertainty of the data falls within 0.01 or 1% error in the measurement of isotope enrichment.

To determine the dimensionality of the isotopomer data, SVD was performed on ^13^C fragments derived from uniformly sampled flux distribution sets for several glucose labels. The results are summarized in Figure 6b. Globally, the choice of glucose labels affects the dimensionality of the resulting isotopomer data set. At the 1% (0.01) threshold, the label with the highest dimensionality was hypothetical [1,2,5-^13^C] glucose with 73 dimensions. The three labels for which experimental data was measured in the subsequent section, [1-^13^C] glucose, [6-^13^C] glucose, and 20% [U-^13^C] glucose, had dimensions 53, 53 and 35, respectively, at this cutoff. These values are all significantly lower than the best label, and, in particular, the uniform labeled experiment only produces half of the dimensionality as the optimal experiment. This result is significant. While 139 dimensions (the number of undetermined dimensions for the model used) are required to specify a unique flux vector, the dimensionality of the ^13^C data for each label is significantly lower. The best labeling experiment specifies just over half (73/139 = 0.52) the degrees of freedom required, and 20% [U-^13^C] glucose only specifies about one fourth of the possible degrees of freedom (0.26). It is worth noting that SVD is a linear operation used to approximate properties of a non-linear system and the true degrees of freedom may be even lower than reported. SVD serves as a useful upper bound on the dimensionality of data for non-linear systems, but the difference between SVD dimensionality and true dimensionality may grow to be unacceptable for large systems. For the system studied here, SVD was found to be of practical use.

### Experimental Validation

In order to assess the agreement of computationally predicted flux elucidation capacity with experimental data, we took fluxomic measurements for three labeling patterns in *E. coli*. Flux distributions that best explain each set of ^13^C data were calculated using a non-linear optimization problem:

$$\begin{array}{c} \underset{v}{\text{min}}\text{Error}\left(v\right)\\ \text{subject}\;\text{to}:\\ v_{min}<v<v_{max}\\ S\cdot v=0 \end{array}$$

The function Error(v) is a score of how well a given flux distribution fits the experimental data. It is defined as:

$$\text{Error(}v\text{)}\;\text{ = }\underset{i\in \;\text{fragments}}{\sum }\frac{{\left(\text{fragmen}{\text{t}}_{i}\left(v\right)-\text{measure}{\text{d}}_{i}\right)}^{2}}{\sigma^{2}}$$

where measured *_i _*is the measured fractional enrichment of fragment *i*, fragment *_i_*(*v*) is the computed fractional enrichment of fragment *i *as a function of the flux distribution *v*, and *α *= 0*:*014 is the standard deviation of the fragments as calculated from experimental replicates.

Reaction flux confidence ranges were then computed for all reactions using all three sets of ^13^C data and all combinations thereof. Confidence intervals for reaction rates were computed by maximizing and minimizing the value of each reaction in turn subject to a slightly relaxed score.

$$\begin{array}{c} \underset{\alpha }{\text{min}}/\underset{\alpha }{\text{max}}c_{i}^{T}\cdot N\cdot \alpha \\ \text{subject}\;\text{to}:\\ v_{min}\leq N\cdot \alpha \leq v_{max}\\ \text{Error (}N\cdot \alpha \text{)}\leq \text{Erro}{\text{r}}_{max} \end{array}$$

where *c_i _*= (0, 0, ...0, 1, 0...0) is a vector of all zeros with a 1 in position i, and *Error_max _*was set based on the confidence value. Because different data sets provide different levels of consistency, Error*_max _*was chosen to be 30 units greater than the minimum error found.

**Figure 7:**
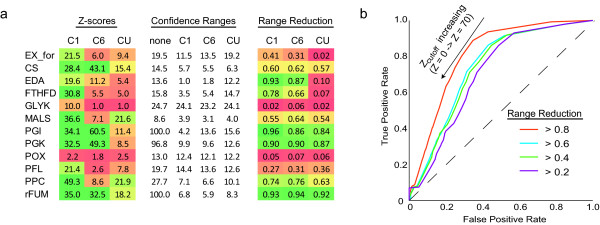
**Comparison of Calculated Z-scores and Experimental Flux Ranges**. a) For several reactions, the computed Z scores are compared to the resulting measured flux ranges. Z-scores show (color coded) Z-scores for each of the 12 reactions and three glucose labels. FVA indicates the absolute allowable flux ranges for three glucoses ([1-^13^C], [6-^13^C], [U-^13^C]) as well as the range if no ^13^C data is imposed ('none'). A normalized version of this table is also presented where all flux ranges are divided by the FVA range thus showing the fraction of flux range remaining. This quantity ranges from 0 (range fully specified) to 1 (no additional information). b) A receiver operating characteristic (ROC) curve for the ability of Z-scores to predict confidence interval reduction by different fractions. Z-scores are observed to predict larger interval reductions (e.g. ratio*<*0.2) with higher specificity and sensitivity than smaller reductions (e.g. ratio*<*0.8).

These intervals were compared with Z-scores calculated through Monte Carlo methods to assess the ability of the Z-scores to predict the size of experimental reaction ranges in a label-specific manner (Figure 7a). The Z-scores were found to be correlated with the relative flux ranges in a statistically significant manner (Student's T-test, p *<*8.6 × 10^-34^). A receiver operating characteristic (ROC) curve suggests that the Z-scores can identify with both sensitivity and specificity the reactions that can be elucidated in a label-specific manner, with better performance predicting ranges that are restricted more by data (Figure 7b). These findings indicate that the Z-score is indeed a useful predictor of the degree a flux range will be constrained by a particular ^13^C experiment and provide experimental support for the computational approach taken.

**Figure 8:**
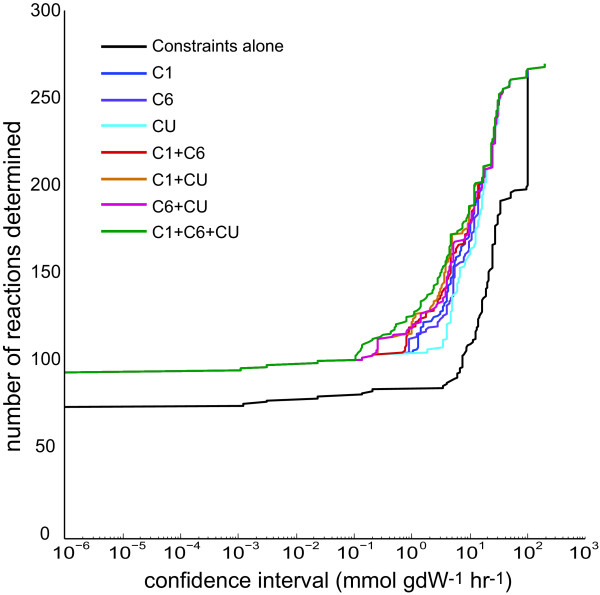
**Reaction Confidence Intervals with ^13^C Data**. The number of reactions determined at a particular confidence given different data is plotted. The range of allowable fluxes (*v_max _*- *v_min_*) were computed for each reaction, constrained by none, one, two or three sets of ^13^C data. Constraints alone are seen to determine a significant number of fluxes, while using all three ^13^C data sets is seen to provide the most elucidated reactions for a particular confidence interval.

The number of reactions elucidated at particular confidence intervals was then found (Figure 8). Using different labels provides different levels of reaction confidence (Additional File 1). Including no ^13^C data generates the largest flux ranges (lower black line), while adding ^13^C data reduces the ranges and shifts the curve left. With almost no exception, including one experiment yields larger confidence intervals than any combination of two carbon sources which in turn is a larger range than including all three sets. Of the single experiment curves, the 20% [U-^13^C] glucose curve provides notably worse ranges than the other two experiments, consistent with the finding that 20% [U-^13^C] substrate provides data with the smallest number of dimensions.

At a reaction confidence of 1 mmol · gDW^-1^·h^-1 ^(a relatively non-stringent cutoff), 85 reactions are specified simply from uptake rate data without any ^13^C data. Performing the least informative ^13^C experiment, using 20% [U-^13^C] substrate, yields 105 reactions that meet the confidence criterion, whereas the combination of all three ^13^C experiments yields 125 reactions that meet the criterion. In other words, performing all three experiments will increase the number of elucidated reactions by 40 reactions or about 50%. As the model used contains 278 carbon-tracking reactions and reaction groups, the increase in knowledge at 1 mmol · gDW^-1^·h^-1 ^confidence from 85 to 125 reactions from using ^13^C data indicates that a large gap in the knowledge remains. It seems apparent that other methods must be developed to obtain flux information at the genome scale from single experiments, as would ultimately be desirable. However, as noted in the above section, the majority of reactions that are elucidated by ^13^C-labeled glucose experiments lie in central metabolic pathways, which tend to be both of high interest and not-specifiable by constraints alone.

## Conclusions

We introduce a new framework for calculating the uncertainty inherent to ^13^C experiments using Monte Carlo Sampling. This allows us to predict the success of experiments before performing them. The method used here 1) does not require experimental identification of the 'real' flux state *a priori *[10] and 2) reports scores for the resolution capability for each reaction as opposed to Boolean identification calls [11]. This framework reveals several key findings:

• The choice of input label is important, as different labels perform better than others. In particular, the commonly-used 20% mixture of uniform label + 80% natural label was shown computationally and experimentally to resolve significantly fewer reaction fluxes than either [1-^13^C] glucose or [6-^13^C] glucose. Thus, the amount of information likely to be obtained by a ^13^C experiment can be predicted in a reaction-specific manner before having to carry out an experiment.

• There is no universally best label. The best label depends on the experimental objective. Certain reactions are more precisely measured with some labels than others, and no label is best at elucidating all reactions. Certain hypothetical ^13^C labels of glucose, for example [1,2,3-^13^C] glucose and [1,2,5-^13^C] glucose, are predicted to perform better than commercially available single labels for many reactions.

• The ^13^C data dimensionality is less than anticipated. Whereas each ^13^C experiment can measure 186 pieces of information at a time, there is a high degree of interdependence. We measured the true data dimensionality to be between 35 and 50 dimensions for commercially available labels and as high as 73 for exotic labels. This high data redundancy can partially explain why ^13^C experiments yield many reaction rates with high uncertainties.

This study suggests limitations of steady-state ^13^C analysis using solely amino acids due to the lower than expected dimensionality of the isotopomer data. However, steady-state ^13^C analysis is clearly still useful elucidating reaction fluxes in *E. coli *metabolism. Notably, as the study was conducted using only protein-derived amino acids, it would be of immediate interest to determine the additional benefit of measuring other classes of labeled metabolites, as well as the benefit of more recently developed experimental techniques such as multi-substrate [10] and dynamic flux labeling [28,29] experiments. Monte Carlo methods are generic and thus are well suited to be adapted to the experimental setup of interest. Additionally, Monte Carlo methods are amenable to biasing the sampling space based on known data to improve results; however, this is expected to incur a corresponding cost in convergence time. Possible sources of error in the current method include unwanted bias in the sampled flux space, possible inadequacy of the Z-score as a statistical metric over more sophisticated tests such as the Kolmogorov-Smirnov test, and inadequacy of the distribution median as a threshold for use in hi-lo hypotheses. Conducting Monte Carlo isotopomer analysis on new systems will become more accessible with the availability of the open source COBRA Toolbox v2.0 for MATLAB, which includes the algorithms presented here [30].

## Methods

### Isotopomer Network Description

The isotopomer network was derived from the iMC1010 *E. coli *reconstruction [19]. The content is reported in the Additional File 1. There are a total of 313 irreversible reactions including 278 that track carbon. All carbon tracking reactions are broken into elementary forward and reverse reactions.

First, a central metabolic isotopomer model was generated that includes a total of 85 reactions, including a biomass production reaction, which drains the precursor metabolites used to make biomass, and 14 system boundary exchange fluxes (for glucose, oxygen, phosphate, NO_2_, NO_3_, acetate, CO_2_, ethanol, formate, fumarate, glycerol, D-lactate, pyruvate, and succinate). The biomass composition is based on one that was reported previously [16,31] and used in the biosynthetic isotopomer model (see details below), but where the biomass components are replaced by the amount of ATP, NADH, NADPH, and central metabolic precursors needed to synthesize the biomass components (Additional File 1). The remaining 70 reactions participate in glycolysis, TCA cycle, pentose phosphate pathway, oxidative phosphorylation, pyruvate metabolism, and anaplerotic metabolism. The central metabolic isotopomer model includes linear mass balance equations for 67 metabolites. Carbon atoms are tracked through 46 metabolites in the core metabolic network. Changes to the central metabolic reactions include assigning fumarate reductase to utilize menaquinone and demethylmenaquinone rather than ubiquinone and adding a phosphate transport reaction coupled to proton symport.

Aside from the central metabolic reactions contained in the central isotopomer model, the biosynthetic model also includes a number of other catabolic and anabolic reactions. The fluxes were calculated with an additional constraint that flux through formyltetrahydrofolate deformylase (which removes the C1 unit from 10-formyltetrahydrofolate) was less than or equal to the measured formate secretion flux. When higher flux through this reaction was allowed the minimum error improved by only 0.3%, but the flux through this reaction was high (around half the glucose uptake rate). To adjust this aberrant behavior, the optimal flux distributions and confidence intervals were calculated with this additional constraint on the formyltetrahydrofolate deformylase flux. The resulting biosynthetic isotopomer model includes 189 metabolites (126 of which have tracked carbon atoms), 313 irreversible metabolic reactions (63 of which are reversible and involve tracked carbon atoms), and 8,612 isotopomer variables (which is equal to the number of non-linear isotopomer mass balance constraints). The model also includes a biomass reaction and 19 system boundary exchange reactions. The biomass reaction was altered to include amino acids, nucleotides, co-factors, and macromolecules rather than their precursor metabolites. In addition, the biosynthetic model balances intracellular protons as well as water molecules similar to iJR904 [16].

### Monte Carlo Sampling

With traditional MCMC, a point is selected within the space which is then iteratively moved around. At each step, a random direction is chosen and the next point is chosen uniformly along this line. The set of points that this algorithm visits will converge to a uniformly distributed set. Two modifications were made: 1) Artificial Centering [32] - Because these biological spaces tend to be elongated in one direction, it is often beneficial to choose directions along the "long" direction rather than uniformly. This can be done by choosing the direction based on previously visited points. At each step, the direction is chosen by drawing a vector from the center of the previous points to one of the previous points chosen at random. 2) In place sampling - Instead of moving just one point throughout the space, many points are moved simultaneously. In this way, no "history" is kept, only the updated position of all the points. This method is described in greater detail in other literature [33,34].

### Computing the Isotopomer Distribution

Each flux distribution and glucose input results in a unique isotopomer distribution. The cumomer method [35] and the elementary metabolite unit (EMU) method [36] were implemented in Matlab and utilized in calculating isotopomer distributions. These methods involve solving several linear systems of equations to compute different groups of isotopomers. For numerical reasons, a routine is introduced which checks whether all parts of the network are still connected at every step. Disconnected components can occur when fluxes to and from the component are zero, making it impossible to compute a unique isotopomer distribution within this subnetwork as many isotopomers satisfy the balance equations. By removing these components first, the other metabolites can be solved in a numerically stable fashion. The resulting isotopomers for the amino acids for each flux distribution are transformed to a mass distribution. This way, each experiment is abstracted to a (number of distributions) × (number of fragments) matrix.

### **Sample Preparation and **^13^**C Measurement**

#### Culture labeling

Prior to labeling, single colonies of *E. coli *K12 MG1655 were selected from stock plates and inoculated directly into 250 ml M9 medium in 500 Erlenmeyer flasks aerated by stirring at 1000 rpm. Cells were grown overnight, harvested, washed twice with water and used to inoculate 50 ml flasks containing 25 ml medium with 2 g/L 13C-labeled D-glucose, with initial OD600 0.005-0.01. Glucose was supplied as either 100% [1-^13^C]-labeled, 100% [6-^13^C]-labeled, or a mixture of 20% uniformly [U-^13^C]-labeled with 80% natural glucose (which is randomly 1% 13C). Cells were grown to mid-log phase, corresponding to OD600 of 0.6. 3 ml of each culture was harvested by centrifugation at 4°C. The media was aspirated and analyzed with HPLC to determine the remaining glucose concentration. Cell pellets were placed at -80°C prior to further analysis.

#### Derivatization and GC-MS analysis

Cells were resuspended in 0.1 ml 6 M HCl and transferred to glass vials. Protein was digested into amino acids under a nitrogen atmosphere for 18 hr at 105°C in an Eldex H/D Work Station. Digested samples were dried to remove residual HCl, resuspended with 75 *μ*l each of tetrahydrofuran and *N-tert*-butyldimethylsilyl-*N*-methyltrifluoroacetamide (Aldrich), and incubated for 1 hr at 80°C to derivatize amino acids. Samples were filtered through 0.2 *μ*m PVDF filters and injected into a Shimadzu QP2010 Plus GC-MS (0.5 *μ*l with 1:50 split ratio). GC injection temperature was 250°C and the GC oven temperature was initially 130°C for 4 min, rising to 230°C at 4°C/min and to 280°C at 20°C/min with a final hold at this temperature for 2 min. GC flow rate with helium carrier gas was 50 cm/s. The GC column used was a 15 m × 0.25 mm × 0.25 m SHRXI-5ms (Shimadzu). GC-MS interface temperature was 300 degrees with 70 eV ionization voltage. The mass spectrometer was set to scan an m/z range of 50 to 600.

#### Processing of GC-MS data

Mass data were retrieved from the GC-MS for fragments of 14 derivatized amino acids: cysteine and tryptophan were degraded during amino acid hydrolysis; asparagine and glutamine were converted respectively to aspartate and glutamate; arginine was not stable to the derivatization procedure. For each fragment, these data comprised mass intensities for the base isotopomer (without any heavy isotopes, M+0), and isotopomers with increasing unit mass (up to M+6) relative to that of M+0. These mass distributions were normalized by dividing by the sum of M+0 to M+6, and corrected for naturally-occurring heavy isotopes of the elements H, N, O, Si, S, and (in moieties from the derivatizing reagent) C, using matrix-based probabilistic methods as described [37,38] implemented in Microsoft Excel. Data were also corrected for carry-over of unlabeled inoculum [37].

### **Computing Reaction Rates from **^13^**C Data**

Reaction rates were computed from ^13^C data as described in the Results. In calculating the best fit flux values from experimental data, a small variation was introduced to reduce the number of variables and remove constraints. Let N be a basis for the null space of *S*. Then all valid fluxes can be written as:

$$\begin{array}{c} v=N\cdot \alpha \\ \underset{\alpha }{\text{min}}\text{Error }N\cdot \alpha \\ \text{subject}\;\text{to}:\\ v_{min}<N\cdot \alpha <v_{max} \end{array}$$

This reduced the number of variables from |*v*| = 335 to |*α*| = 139. Optimization was performed with the Tomlab/SNOPT package. This method is an iterative local optimization and is therefore not guaranteed to find the optimal solution. To address the issue of local minima, the procedure was run with many randomly generated starting points and the lowest minimum was taken.

### Code and Equipment

The code was written in the MATLAB environment and the COBRA toolbox. Linear Programming was done with the Tomlab/CPLEX package and nonlinear optimization with the TOMLAB/SNOPT interface. The EMU and cumomer method were written in native Matlab but generated in Perl. Computations were performed on a Dell Studio XPS desktops (2.6 Ghz core i7 with 9-12 GB ram) and a custom Rocks cluster (100 dual Xeon 5500 series nodes).

## Authors' contributions

JS devised methods and drafted manuscript. DZ performed additional analysis and completed manuscript. WC and SM implemented methods in Matlab and performed computational analyses. VP grew *E. coli *samples. DS performed MS fragmentation and natural abundance correction. JLR generated the ^13^C *E. coli *model with ^13^C mappings. AO and BOP were responsible for the strategic vision. All authors have read and approved the manuscript.

## Supplementary Material

Additional file 1 **Z-scores, confidence intervals, and isotopomer model**. This file contains the absolute and relative Z-scores for individual reactions across all glucose labeling patterns tested, confidence intervals calculated using experimental ^13^C tracing data, and details of the model that was used in calculations. Click here for file
